# Assessing community readiness for early intervention programmes to promote social and emotional health in children

**DOI:** 10.1111/hex.12887

**Published:** 2019-04-10

**Authors:** Shahid Islam, Neil Small, Maria Bryant, Sally Bridges, Nicola Hancock, Josie Dickerson

**Affiliations:** ^1^ Faculty of Health Studies University of Bradford Bradford UK; ^2^ Faculty of Health Studies University of Bradford Bradford UK; ^3^ Diet, Obesity & Lifestyle Portfolio lead, Clinical Trials Research Unit Leeds Institute of Clinical Trials Research, University of Leeds Leeds UK; ^4^ Better Start Bradford Innovation Hub, Born in Bradford Bradford Royal Infirmary, Bradford Teaching Hospitals NHS Foundation Trust Bradford UK; ^5^ Better Start Bradford Mayfield Centre Bradford UK

**Keywords:** community readiness model, deprived neighbourhoods, early intervention, parenting interventions, pregnancy and maternity, social and emotional health

## Abstract

**Objective:**

Evidence for early intervention and prevention‐based approaches for improving social and emotional health in young children is robust. However, rates of participation in programmes are low. We explored the dynamics which affect levels of community readiness to address the issues of social and emotional health for pregnant women, young children (0‐4 years) and their mothers.

**Setting:**

A deprived inner‐city housing estate in the north of England. The estate falls within the catchment area of a project that has been awarded long‐term funding to address social and emotional health during pregnancy and early childhood.

**Methods:**

We interviewed key respondents using the Community Readiness Model. This approach applies a mixed methodology, incorporating readiness scores and qualitative data. A mean community readiness score was calculated enabling the placement of the community in one of nine possible stages of readiness. Interview transcripts were analysed using a qualitative framework approach to generate contextual information to augment the numerical scores.

**Results:**

An overall score consistent with vague awareness was achieved, indicating a low level of community readiness for social and emotional health interventions. This score suggests that there will be a low likelihood of participation in programmes that address these issues.

**Conclusion:**

Gauging community readiness offers a way of predicting how willing and prepared a community is to address an issue. Modifying implementation plans so that they first address community readiness may improve participation rates.

## 1 INTRODUCTION

Early intervention programmes (EIP) during pregnancy and for parents of young children hold considerable promise for the development of children's social and emotional health.^1^, ^2^, ^3^ These programmes include interventions that encourage positive parenting skills or that reduce the risk of perinatal depression.^4^, ^5^ The quality of interactions in the first few years of life can affect a child's life course trajectory well into adulthood.^6^, ^7^, ^8^ In consequence, early intervention policies and programmes which enable parents to help children achieve the best start in life are promoted as a blueprint for a healthy and happy society.^9^, ^10^, ^11^, ^12^, ^13^

Social and emotional health is a multifaceted concept encompassing the development of self‐control, building relationships and learning skills and abilities to help children succeed in school and broader society.^14^ NICE guidance makes a point that—“good social, emotional and psychological health helps protect children against emotional and behavioural problems, violence and crime, teenage pregnancy and the misuse of drugs and alcohol”.^14^ Subsequent guidance concurs and adds “happiness and confidence” as outcomes of positive social and emotional well‐being which can act as protective factors against depression.^15^

Early intervention is vital as the optimum time to influence developmental trajectories recedes with age.^16^ Perinatal depression provides a lucid example; mothers experiencing perinatal depression can adversely impact on children's emotional and cognitive health because this type of depression “coincides with a period of substantial baby brain development during which infants are entirely dependent on their primary caregivers for physical care, security, and emotional regulation”^17^ (p608). Missed opportunities during the formative years of a child's life could contribute to a disadvantaged adulthood,^17^, ^18^, ^19^ something personally damaging and distressing as well as costly for government and society.^20^, ^21^, ^22^ This is a context that has given rise to a wide range of approaches including family support services, parenting programmes and one‐to‐one support for expectant and new parents during pregnancy and the first years of a child's life.

The extant literature broadly supports EIP approaches to address issues relating to children's social and emotional health.^9^, ^23^, ^24^ Particularly in contexts of social disadvantage, parenting courses can contribute to positive child outcomes such as improved school readiness and enhanced rates of academic success.^25^ Moreover, the social and developmental benefits can be felt as much as 20 years after attendance.^8^ Parents support the principle of early intervention to enhance social and emotional health development, especially parents in lower socio‐economic groups.^26^, ^27^ But across all socio‐economic groups, there appears to be a difference between expressed favourable support which is high, and uptake, which is often low. For example, Cullen and colleagues^28^ asked parents of young children how likely they were to participate in parenting classes and found that 33% said they *might participate *and 10% said they *were likely *to participate; however, when take‐up data were examined at the end of a pilot of free parenting classes, only 6% of eligible parents had taken part. This gap between *intention* and *action* is what prompted Daro and McCurdy^27^ to note in relation to uptake of family support programmes that—“what people say and what they do is qualitatively different” (p.115).

Whilst research has been predominantly concerned with individual and family influences, parenting and children's social and emotional health are also impacted by neighbourhood factors. Neighbourhood poverty can impede the quality of parenting.^29^ Issues that stem from living in a highly deprived neighbourhood include increased stress 30, 31, 32 and higher numbers of lone parent households with subsequent pressures on parental time.^33^, ^34^ Areas with high rates of ethnic diversity may experience higher inequities as research has shown women from minority ethnic backgrounds can be twice as likely as White British women to miss detection of common mental disorders.^35^ This omission is a risk factor in terms of identified socio‐emotional and behavioural difficulties in their young children.^32^

Given research evidence of effectiveness and parental enthusiasm for EIP but recurring challenges in recruitment to programmes, the aim of this study was to examine levels of community readiness amongst residents living in a deprived neighbourhood to participate in a programme which aims to enhance and address issues related to social and emotional well‐being for young children. We report our findings after the application of the community readiness model (CRM); a key principle of the CRM is “that unless a community was ready, initiation of a prevention programme was unlikely, and if a program started despite the fact the community was not ready, initiation was likely to lead only to failure”^36^ (p.293). To our knowledge, the CRM has not hitherto been applied and findings published after gauging readiness for social and emotional health issues.

## 2 METHODOLOGY

The CRM is a practical toolkit which seeks to provide some approximation of the likelihood that a community will engage and participate in a programme designed to address a specific issue. The model was originally developed in the United States for assessing a community's readiness to address alcohol and drug abuse and has subsequently been applied to cover a broad range of issues including obesity prevention, HIV prevention and deforestation issues.^37^, ^38^

### 2.1 The CRM tool

The CRM is a mixed method approach which incorporates a qualitative component^39^, ^40^ and a numerical score. The CRM comprises of 36 questions spread across six dimensions of readiness, these are as follows: community efforts, community knowledge of the efforts, leadership, community climate, community knowledge of the issue and resources for prevention (see below for examples of questions). The model identifies nine stages of readiness that range from “no awareness” of the issue to “high level of community ownership” (see Table 1). Once a community's stage of readiness is identified, plans can be formulated to raise levels of community readiness through engagement and communication exercises appropriate at each level and barriers that may impede community participation can be addressed.

**Table 1:** Nine‐point readiness scale for Community Readiness Model

Stage	Description
1. No awareness	Issue is not generally recognized by the community or leaders as a problem (or it may truly not be an issue)
2. Denial/resistance	At least some community members recognize that it is a concern, but there is little recognition that it might be occurring locally
3. Vague awareness	Most feel that there is a local concern, but there is no immediate motivation to do anything about it
4. Pre‐planning	There is clear recognition that something must be done, and there may even be a group addressing it. However, efforts are not focused or detailed
5. Preparation	Active leaders begin planning in earnest. Community offers modest support of efforts
6. Initiation	Enough information is available to justify efforts. Activities are underway
7. Stabilization	Activities are supported by administrators or community decision‐makers. Staff are trained and experienced
8. Confirmation/expansion	Efforts are in place. Community members feel comfortable using services, and they support expansions. Local data are regularly obtained
9. High level of community ownership	Detailed and sophisticated knowledge exists about prevalence, causes and consequences. Effective evaluation guides new directions. Model is applied to other issues

### 2.2 Ethics

Ethical approval for this study was granted by the University of Bradford Ethics Committee on 22 December 2016 (EC2435).

### 2.3 Setting

We applied the CRM to a housing estate which, according to the National Indices of Multiple Deprivation mapping software, is split into two super‐output areas: placed at position 104 and 134 out of a possible 32 844 super‐output areas in the UK.^41^ Lower scores are indicative of higher levels of deprivation and so this places our estate amongst the 150 most deprived UK neighbourhoods. The housing estate is highly diverse in terms of ethnicity with a mixture of White working‐class households and minority ethnic communities many of which are of Pakistani heritage with more recent arrivals coming from Eastern European countries. The community is located within a catchment area of the Better Start Bradford programme, a 10‐year Big Lottery funded initiative to deliver interventions to address a range of health and social disparities affecting children's development.^10^, ^42^ The interventions delivered to support the development of social and emotional health for children include support for teenage mothers, a befriender scheme for all mothers affected by or at risk of post‐natal depression, healthy lifestyle advice and a range of targeted and universal parenting programmes.

### 2.4 Recruitment and consent

A date and venue were arranged for each interview during the invitation telephone call with participants. All participants were asked to provide informed consent prior to any data collection.

### 2.5 Participants

The CRM relies on interviewing between four and six local key respondents who understand the community in an esoteric way,^36^, ^43^ for example community leaders or community activists. Purposive sampling was used to identify potential respondents through discussions with a Community Research Advisory Group (CRAG) which is an established and ongoing group comprised of members of the public who live in the Better Start Bradford area. The group was set up to consider research issues from the vantage point of communities. Key considerations of this group include appropriateness and acceptability of research methods and questions.

Community Research Advisory Group members were able to identify key respondents who were well placed to answer the questions listed in the CRM for the aforementioned issues and area. On the subject of sampling, the CRM handbook advises—“try and find people who represent different segments of your community” 43 (p10) and then offers a list of who could be included. With this in mind and with the advice taken from CRAG, we aimed to recruit a minimum of six community leaders/key stakeholders. Eight individuals were invited to take part via telephone. However, two potential participants were not eligible; both represented faith organizations (Mosque and Church) but informed us that most worshippers came from outside the eligible area. This notwithstanding, we were able to recruit six key respondents. This is an acceptable number of participants necessary to complete the assessment 43 and equates with the findings from a systematic review 37 which reported a similar number of key respondents were recruited by other CRM studies (mean = 7.3; median = 6).

Our sample included key respondents from a diverse range of backgrounds including Rohail and Laura who were, respectively, employed and volunteered with non‐profit organizations to support and engender community activism through a range of methods including community clean‐ups, residents associations and liaison with schools. We had three local authority employed professionals who were highly active in the neighbourhood and contracted by different statutory organizations: the children's centre (Fazal), the primary school (Jason) and the neighbourhoods team (Katrin). Our final key respondent was a ward councillor who represented the ward in which the estate is located on the city council (Ali). All names used in this paper are pseudonyms.

### 2.6 Data collection

Interviews were conducted by the first author in community locations which were most convenient for respondents and took place during a four‐month period from February to May 2017. Interviews began with a discussion to clarify the meaning of the term “social and emotional health.” To facilitate this discussion, key points relating to social and emotional health, as defined by NICE guidance,^15^ were explained to the respondent. The subsequent discussion varied because levels of familiarity and awareness about this issue differed between key respondents. We sought to ensure a commonly agreed meaning of this term existed across all respondents.

### 2.7 Topic guide

Interviews used the topic guide found in the handbook of CRM.^43^ This included 36 questions relating to the six dimensions of the tool. By way of example, the open‐ended questions included the following:

What type of information is available in your community regarding this issue?
What does the community know about these efforts or activities?
How are these leaders involved in efforts regarding this issue? Please explain?

### 2.8 Analysis

Interviews lasted between 34 and 68 minutes and were audio‐recorded and transcribed verbatim. Respondent's transcripts were given a pseudonym to ensure confidentiality, and their job roles removed to reduce the risk of identification. Interview transcripts were independently scored by two authors (NH and SB) trained in using the anchored rating scales of the community readiness model to assign scores ranging from one to nine for each of the six dimensions. Following the guidance for completing a community readiness assessment,^43^ both scorers independently rated each of the six interviews and then agreed a consensus score for each interview after discussing and resolving differences in scores they had independently reached. The consensus scores were then summed across each dimension and divided by the number of interviews to generate a mean stage score for each of the six dimensions. The scores ranged from *one* (no awareness) to *nine* (community ownership). The dimension scores and the overall mean community score are rounded down, as per the guidance.^43^

Some commentators (notably Kesten and colleagues^40^) highlight the importance of using the qualitative data generated through the application of CRM to understand context and score as, they argue, these are inextricably linked. We therefore analysed the qualitative data through NVIVO 11 software using framework analysis.^44^, ^45^ This was completed by the first author (SI) with supervision and support provided by the second author (NS). Since the purpose of this study was to produce a useful categorization scheme for community readiness using questions organized around the six dimensions, we then arranged these *a priori* dimensions into analytical themes. These were indexed systematically, a process which entailed comparison within and between the themes. As the analysis evolved, it became necessary to chart and rearrange segments of the data to ensure contents were placed under the heading of the theme that was most appropriate. For example, when issues discussed under the theme of *knowledge about efforts* seamlessly segued into discussions about *community climate *then these were appropriately relocated.

### 2.9 Data validation

The qualitative analysis was validated through discussion with two authors (NH and SB) who were familiar with the transcripts and findings. Data interpretations were also discussed within the wider research team who were able to provide guidance about the emergent findings. After completion, key respondents were emailed a short report which included the numerical scores along with a summary of key findings. This email was accompanied with an invitation to contact two members of the research team in case further clarification would be helpful (NH and SB). This process served two purposes. Firstly, closing the feedback loop through *debriefing* is an important part of conducting ethical research,^46^ and second, a useful yardstick, according to Greenhalgh,^47^ by which to measure validity from qualitative research findings is to ask “how comprehensible would this be to a thoughtful participant in the setting?”(p.176). We followed these steps and key respondents let us know they were thankful for receiving the findings, though no queries were returned.

## 3 FINDINGS

Topical points and verbatim quotes presented below will draw on the salient issues raised during interviews to help us comprehend the numerical scores achieved across the dimensions. As there were several areas of overlap between the dimensions (eg between attitude of leaders to the issue and how this affects resources available for prevention), it has been necessary to present the results in an aggregated way rather than treating each dimension as an independent unit.

### 3.1 Overall CRM score

The mean overall CRM score was three (SD = 1.17), corresponding with *vague awareness* stage of readiness for social and emotional health. The mean scores for each dimension (Table 2) varied, ranging from *two* to *five* which suggests that, for this community, some dimensions displayed more readiness than others. The overall score of *three* is described in the following way by the authors of the CRM:

There is a general feeling amongst some in the community that there is a local problem and that something ought to be done about it, but there is no immediate motivation to do anything. There may be stories and anecdotes about the problem, but ideas about why the problem occurs and who has the problem tend to be stereotyped and/or vague. No identifiable leadership exists or leadership lacks energy or motivation for dealing with this problem. Community climate does not serve to motivate leaders 36 (p.298)

**Table 2:** Neighbourhood community readiness scores for social and emotional health

Dimension	Interviews						Mean (SD) stage score
	1	2	3	4	5	6	
Community efforts	4.00	4.00	6.25	7.00	7.00	2.25	5 (1.95)
Community knowledge of the efforts	4.50	1.50	4.25	3.00	3.00	3.00	3 (1.08)
Leadership	3.75	4.50	3.75	3.25	6.50	4.00	4 (1.16)
Community climate	1.75	1.75	2.50	3.00	2.25	1.75	2 (0.52)
Community knowledge about the issues	2.75	3.25	3.25	2.25	2.25	2.50	2 (0.46)
Resources related to the issue	4.25	4.75	4.35	6.00	4.00	5.00	4 (0.72)
Overall CRM score							3 (1.17)

### 3.2 Community efforts and Knowledge about efforts

The highest score was found in the *Community effort* dimension (*five* SD 1.95) which indicates that it was believed efforts had been made to improve issues around social and emotional health, but these did not necessarily translate into the community attaining sufficient *knowledge about these efforts* as that dimension scored *three* (SD 1.08). This difference indicates that a mismatch may be present between available provision and residents' awareness about what is on offer. We found levels of awareness were bound up with a range of other variables, as described by Katrin:

“Well, I guess it depends on how connected they are to the community and its services. If you're somebody that's lived in the area a long time, you speak very good English, you know what's on offer, you're not frightened about asking questions, then you'll know a lot. If you go to somebody else who has quite a sheltered life and doesn't have any children at the school, doesn't really get involved in anything much outside the house, then they're not likely to know what's going on and what's available” (Katrin).

### 3.3 Community knowledge about issues

*Knowledge about current efforts *(ie what is currently available as discussed above) and *knowledge about issues* are crucially different, which is why they are recorded and scored separately in the CRM. The former concerns itself with trying to understand how much residents know about what is locally available in relation to the issue, whilst the latter is concerned with the question of—are people aware *why* the issue is important. This latter dimension attained a low score of *two* (SD 0.46) which is consistent with the denial/resistance stage. The two quotes below contextualize the numerical score:

“A lot of them wouldn't even realise they've got these problems. Denial, not wanting to face up to it”. (Laura)

“Well, I think one of the main weaknesses is about talking about these sorts of things. You have to admit that you have an issue and a problem, and even if you do, is there any point talking about it if there's no solution to it?” (Katrin).

We anticipated some difficulty in achieving a consensus on what is meant by social and emotional health. In part, that difficulty is because social and emotional health issues are not as corporeal to describe, as say, obesity or drug abuse (topics explored in previous studies using the CRM). Difficulties can also arise because whilst we were concerned with social and emotional health of children and their parents, respondents might wish to reply with thoughts that better relate to these issues in the neighbourhood for everyone. Our concerns were partially realized. Half of our respondents were able to offer vivid examples about problematic social and emotional health for children and mothers and the associated consequences with a degree of confidence. These were respondents who were employed in roles that had social and emotional health development as a significant component of their work. The remaining half, who were less familiar with the lexicon of social and emotional health (eg attachment disorders, conduct disorders and post‐natal depression), instead focussed on neighbourhood concerns connected to social deprivation (eg crime, domestic violence and poverty) and described how variables such as these impeded positive social and emotional development for everyone.

Despite these epistemic differences and varying levels of familiarity with the issues, respondents converged on two crucial points that may explain a lower level of awareness, and perhaps, a lower likelihood of uptake in projects. Firstly, social and emotional health problems in children may not be immediately apparent in terms of behavioural changes. This could make it difficult for parents to know whether help should be sought early. Problems whose origins are in early childhood may become manifest as a concern some years later. Secondly, for mothers, there is not a direct relationship between experiencing mental health problems and the decision to seek help. Katrin, for example, describes what would happen if a mother was experiencing lowered mood:

“So you wouldn't necessarily think, "Oh well, I need to get some help with my wellbeing, where can I go? It's not a physical problem, what can I do? And maybe I'll just buck up in few days or whatever". (Katrin)

### 3.4 Community climate

The interview with Jason allowed us to segue from the above topics into the theme of community climate as he saw the issue in a nuanced and dynamic way:

“I don't think they (residents) just see it as one issue, that's the first thing. I don't think, if you said to them, you know, what are your thoughts on social and emotional health, I don't think they'll just see it as one issue. There's so many factors intertwined with this one. I know we're only talking about this today but it's very difficult just to talk about this on its own”.

All of the respondents drew a link between poverty and social exclusion and how that could lead to the negation of health and well‐being matters in order to concentrate on pressing concerns such as seeking employment and paying food and fuel bills. High levels of deprivation and antisocial behaviour and crime often coexist in the same neighbourhood,^25^, ^30^, ^48^ and these were mentioned as part of the daily reality for many of the children growing up in this neighbourhood. Comments included the following:

“Some of these parents have been through traumatic times and it's had a massive impact on their child. Some of the parents have got drug issues and it's normal to these children”. (Laura)

“Domestic violence is quite a big issue round here – bigger than other places” (Fazal)

“I think the challenges that are in [this estate], sort of socio‐economic challenges that exist for the people there, you know, a lot of lone parents, many people on benefits, low education attainment, high crime rates, lots of drug use, abuse, drug dealing, very few clean and safe places to play”. (Rohail)

The *community climate* dimension, for these reasons, scored *two *(SD 0.52) which is indicative of the denial/resistance stage. But this should not imply a sense of apathy or hopelessness as we were informed, on the contrary, people in the neighbourhood felt a sense of social solidarity and community spirit which was often misunderstood and misrepresented by outsiders to the area. Rohail was trenchant on this point when he told us:

“The people of [this estate] have had to build resilience out of, which has been born out of struggle and hardship because they've never benefitted from the New Deal funding which was from 2000 to 2010. And they've had to go at it alone” (Rohail).

Later in the interview he told us:

“There's a lot of volunteering that goes on in the area, you know, there's a lot of civicness that happens, that goes un‐noted” (Rohail).

### 3.5 Leadership and resources

These points are of crucial importance to the dimensions of *Leadership* and *Resources* for prevention, both of which achieved a score of *four* (SD 1.16 and SD 0.72, respectively), consistent with the pre‐planning stage of the community readiness model. This is an improvement on the overall score of *three* and that improvement reflects how we heard leaders would provide support to ideas and would welcome resources that would improve social and emotional health. However, as the earlier comments allude, there are low levels of expectations within the community that any help will be forthcoming. Katrin expanded on this by highlighting the inherent difficulties when decisions are taken by funding bodies:

“I know that people are interested in trying to do more for the area, I know that the Community Council are looking to include [the estate] in their area because they see that there's lot of need in that area, but it's also, I think, frightening for them because they have very limited funds at their disposal and they see that it could all get swallowed up [here]. I think also the Ward Councillors are quite concerned and they do see that it's an issue, but maybe, you know, not one that they're able to devote much effort into thinking about, and they've got other parts of the ward that also have issues”.

This quote illustrates that whilst leaders wish to achieve more for the neighbourhood, they are equally reluctant to translate this ambition into any meaningful action because this may take more resource and effort than what is currently available. Relative deprivation theory predicts that a disadvantaged neighbourhood may be more supportive for low‐income residents than a mixed neighbourhood 48 and evidence to support this theory was found in responses to our question—*who do people turn to for help and support with issues related to social and emotional health?* Consider the following:

“Initially they probably start with, or start off with, their friends and families I reckon”. (Fazal)

“Probably to neighbours and within their own community, within, you know, friends, family members, that sort of stuff” (Rohail)

“I guess it would be another family member by and large, or a friend”. (Katrin)

“There's a lot more supporting each other now. Everybody has got friends from all the communities and they are willing to help each other”. (Ali)

Laura held a different view and was categorical that most people in the area would struggle to face up to their problems, “because for me people aren't going to be truthful and say yes, I've got this problem because of fear of getting their children took off them”. When Laura was probed further—who would residents turn to if the need was urgent?—her response was resolute:

“They probably wouldn't. They'd wait for somebody to go to them, you know, somebody that they can trust and… I think a lot of people are in denial” (Laura).

Jason told us that the local school and nursery provide an outlet that parents could trust. He made it clear that achieving this level of trust had involved a concerted effort:

“I mean, we've just received an “Engaging Families” award because of how effective we are engaging our families so we have a medium, we have very different mediums of communication. Communication is essential to this so we have a text messaging service, we have newsletters, we have week, fortnightly newsletters, we have curriculum newsletters, half‐termly, we have parent ambassadors, we have staff on the gates every morning, every evening informing parents, we have a six weekly parent forum, we have parent consultations biannually (…) we believe we are very, very good at engaging our parents and informing them what goes on. The nursery is quite good as well, they've got people on the door, on the gates. (…) on a Tuesday morning because you just have to see the line, the queues outside there on a Tuesday morning and that tells you”.

The numerical findings for the six dimensions have enabled us to visualize the complex web of elements of community readiness, and the interview dialogues have shown us how the strands that make this web were weaved. When both forms of data, quantitative and qualitative are placed side‐by‐side, they offer a plausible level of predictability about the state of preparedness in a community to address a specific concern.

## 4 DISCUSSION

Based on the collective dimension scores using the Community Readiness Model, our neighbourhood was deemed to be at a stage of vague awareness (*three*) and, as such, showed low levels of community readiness to address issues related to social and emotional health. We saw the availability of services did not match awareness about those services, and we identified a gap in scores between community efforts (*five*) and knowledge about the issues (*two*). Making services available does not mean people will access them.

Research from the early 1980s^49^ highlighted that mothers in high‐density support networks were more likely to refuse parenting services than mothers with fewer social supports. Similar findings were noted by research which highlighted that people from working‐class backgrounds and ethnic minority groups were more likely to turn to people in their social networks for help with parenting support and therefore less likely to access services compared to families in affluent areas.^50^ Some commentators (notably Daro and McCurdy^27^) attribute this differential in seeking support to a cost and benefit calculation that families do; “mothers who have abundant support perceive fewer program benefits to offset the potential costs of involvement than do mothers who are raising children with limited help from others” (p.115). Our findings, however, posit that low levels of participation are also explained by variables including knowledge about efforts, knowledge about issues and the overall community climate.

A notable strength in applying the community readiness model is the ability to independently analyse its various domains. This allows plans to be formulated to tackle each domain in its own right. For example, a low level of awareness about the issue would necessitate a communication plan to enable residents to see the value of seeking help and to recognize the symptoms and circumstances that indicate when help should be sought. It would not be sufficient, in this instance, to make families aware about existing services (ie when a programme is next scheduled) if people have no prior knowledge about how the issue may affect their family life and what benefits may be accrued through participation.

There are some limitations in applying the CRM method. Key amongst these is the emphasis on key respondent views. This means that an understanding of community readiness is at best a proxy measure that bypasses the people who are most likely to be affected by the interventions—the residents of the target area. Whilst the CRM handbook^43^ does encourage the inclusion of community voices, it does not go as far as including them in determining the overall community readiness score. The method could be modified or augmented, broadening the definition of key respondents perhaps and using other qualitative methods to seek resident views on barriers and facilitators to their participation.

Whilst this housing estate may share many characteristics with other estates in the UK such as social deprivation, ethnic diversity and social exclusion,^33^, ^51^ this is not sufficient to assume the same score will be attained in another neighbourhood even if there is a strong demographic resemblance. This is because dimension scores will vary according to levels of efforts made to deliver services coupled with how engaged the community has been about the issues. Dimension scores vary within a community (as we saw through this research), and they are just as likely to vary across communities.

Poverty, social exclusion and the dimensions we have discussed coalesce in a way that can influence community readiness. For example, it was suggested that residents were more likely to be pre‐occupied with issues such as lone parenthood, domestic violence, fear of crime and safety in their neighbourhood rather than with seeking ways to improve social and emotional health of young babies and pregnant mothers.

A community climate which is dominated by deprivation plays a significant role in relegating seemingly non‐urgent issues, from a day‐to‐day parenting perspective to the lower end of a community's priorities. This presents programme implementers with a dialectical dilemma whereby poverty and concomitant social exclusion lead to a reduced likelihood of participation in EI programmes (such as parenting classes), but enrolment in such programmes can potentially improve children's life course trajectories and ameliorate poverty for future generations. This predicament carries echoes of Dr Samuel Johnson's observations more than 200 years ago when he wrote about poverty and said it “certainly destroys liberty, and makes some virtues impracticable and others extremely difficult” (p.141).^52^ Similar impediments help us understand why it might be problematic today for families living in poor neighbourhoods to attend programmes even though they offer hope for children to achieve positive social and emotional development.

## 5 CONCLUSION

This paper shows that applying the CRM methodology to an important issue in a dynamic community can provide insight on why a community may not embrace a programme despite its robust evidence base and potential to improve children's social and emotional well‐being. The CRM is able to identify, at a granular level, the domains that can be addressed to enhance levels of community readiness. This approach can enable policymakers and service providers to work in harmony with the level of community readiness, thus maximizing chances of successful implementation.

## INFORMATIVE

Early intervention programmes targeted at families with young children to improve social and emotional health are promoted widely especially in neighbourhoods with high levels of deprivation. Evidence shows that whilst rates of promotion are high, participation is generally low. This study explores what impact community readiness may have on levels of preparedness amongst residents living on a local authority council estate.

## CONFLICT OF INTEREST

Nicola Hancock is employed by Better Start Bradford and was involved in the scoring process. At the time of fieldwork or during the scoring process, Nicola was not responsible for the work commissioned or planned by Better Start Bradford to address social or emotional health‐related issues. None of any of the other authors have any conflict of interest to declare.
